# Digital technology and mental health during the COVID-19 pandemic: a narrative review with a focus on depression, anxiety, stress, and trauma

**DOI:** 10.3389/fpsyt.2023.1227426

**Published:** 2023-12-22

**Authors:** Paul C. Guest, Veronika Vasilevska, Ayoub Al-Hamadi, Julia Eder, Peter Falkai, Johann Steiner

**Affiliations:** ^1^Department of Psychiatry, Otto-von-Guericke-University Magdeburg, Magdeburg, Germany; ^2^Laboratory of Translational Psychiatry, Otto-von-Guericke-University Magdeburg, Magdeburg, Germany; ^3^Laboratory of Neuroproteomics, Department of Biochemistry and Tissue Biology, Institute of Biology University of Campinas (UNICAMP), Campinas, Brazil; ^4^Department of Neuro-Information Technology, Institute for Information Technology and Communications Otto-von-Guericke University Magdeburg, Magdeburg, Germany; ^5^Department of Psychiatry and Psychotherapy, University Hospital Ludwig-Maximilians-University Munich, Munich, Germany; ^6^Center for Health and Medical Prevention (CHaMP), Magdeburg, Germany; ^7^German Center for Mental Health (DZPG), Center for Intervention and Research on Adaptive and Maladaptive Brain Circuits Underlying Mental Health (C-I-R-C), Halle-Jena-Magdeburg, Magdeburg, Germany; ^8^Center for Behavioral Brain Sciences (CBBS), Magdeburg, Germany

**Keywords:** COVID-19, depression, PTSD, anxiety, APP, chat bot, electronic diary, gameplay

## Abstract

The sudden appearance and devastating effects of the COVID-19 pandemic resulted in the need for multiple adaptive changes in societies, business operations and healthcare systems across the world. This review describes the development and increased use of digital technologies such as chat bots, electronic diaries, online questionnaires and even video gameplay to maintain effective treatment standards for individuals with mental health conditions such as depression, anxiety and post-traumatic stress syndrome. We describe how these approaches have been applied to help meet the challenges of the pandemic in delivering mental healthcare solutions. The main focus of this narrative review is on describing how these digital platforms have been used in diagnostics, patient monitoring and as a treatment option for the general public, as well as for frontline medical staff suffering with mental health issues.

## 1 Introduction

The COVID-19 pandemic has had catastrophic effects on physical health and mortality with over 670 million confirmed cases and 6.8 million deaths worldwide as of March 3, 2023 (1). The consequences of the pandemic on mental health have also been devastating with increases or exacerbations seen in disorders such as depression, anxiety and post-traumatic stress disorder (PTSD). This has occurred due to the social isolation, lockdown and other governmental measures taken to stop the spread of the virus (2–4). In addition, it is now apparent that a significant proportion of these cases may be a direct consequence of viral infection or post-infection neurological sequelae (5–8).

**Figure 1:**
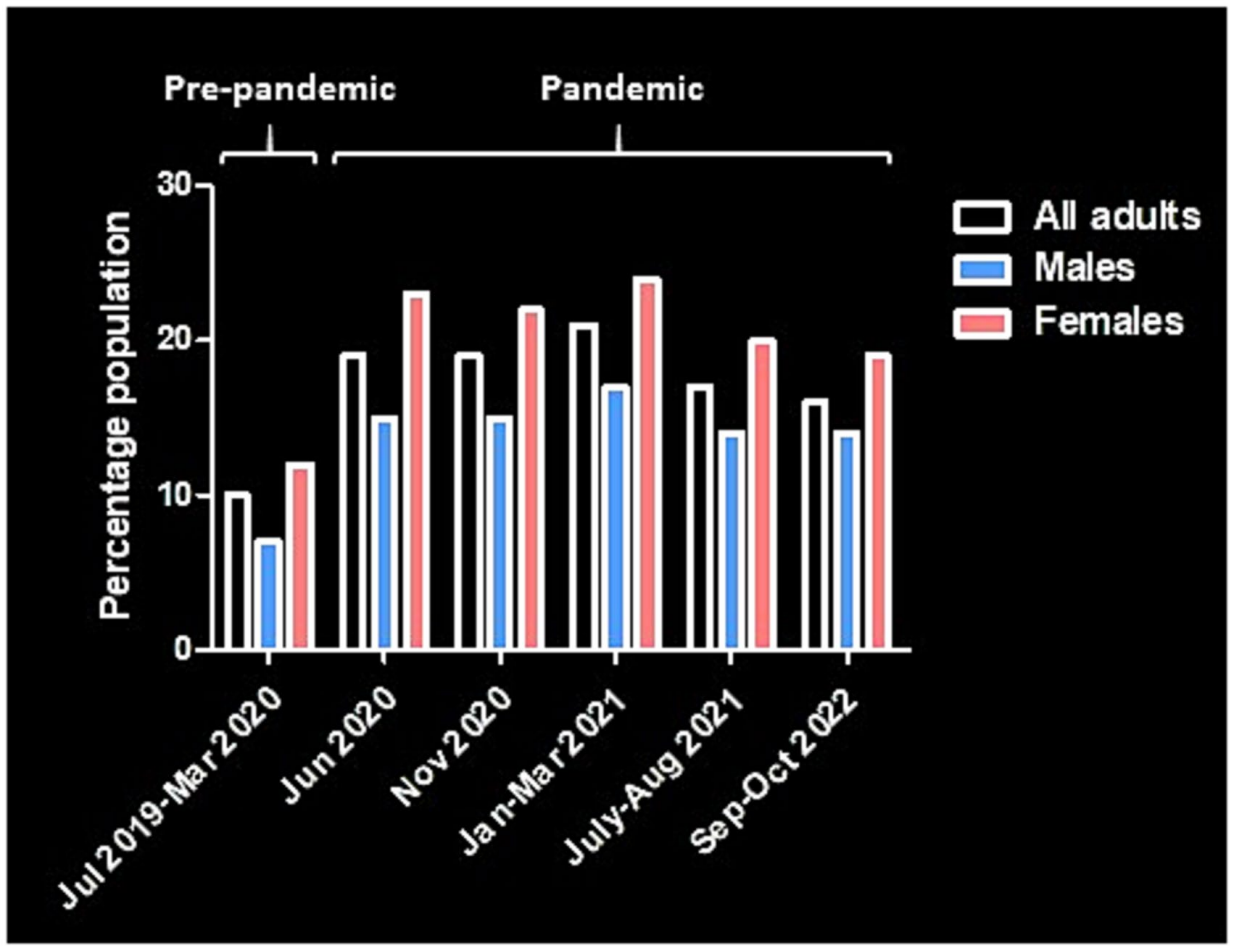
Diagram showing the prevalence of depression in adults in the United Kingdom before and during the COVID-19 pandemic [data from (9, 10)].

According to the Office for National Statistics in the United Kingdom (UK), an estimated 10% of the adult population had moderate to severe depressive symptoms during the period of July 2019 to March 2020, before the COVID-19 pandemic and ensuing lockdowns and social restrictions began (9). This percentage approximately doubled to 19% by the first 3 months of the first COVID-19 wave, peaked at 21% during January to March 2021, and marginally eased to 17% by August 2021 and to 16% by October 2022 (Figure 1) (9, 10). Despite these effects on mental health, fewer people with depression and other mental disorders sought help from healthcare professionals during the first 18 months of the pandemic (11). The waiting time for psychotherapy amounted to several months even before the pandemics. According to the German Psychotherapists’ Association, the average waiting time reached 19 weeks in 2017 (12). Because many digital apps are practitioner-independent, the use of the apps provides faster help for patients seeking psychotherapy, while guided apps are more effective (13).

Depression and other depressive disorders are often managed by a general practitioner, although more severe cases may require specialist services (14). Most of the time, depression is treated with antidepressants or psychotherapy, although these approaches are not always successful. In England, the Royal College of Psychiatrists estimated that an improvement in symptoms with the first antidepressant prescribed occurs for only 50–65% of patients and unsuccessful cases may be prescribed other antidepressants in a successive manner until symptom improvements are achieved (15). Thus, a high proportion of patients can go several weeks or months without any signs of improvement (16). This can create a significant burden at the individual, healthcare and societal levels, and highlights the importance for improvement of existing approaches or development of new treatment strategies for this debilitating mental disorder. Furthermore, this situation was compounded by reduced access to both outpatient and inpatient mental healthcare during heightened periods of pandemic where lockdown and social restrictions were at their highest. Thus, efficient solutions were needed to cope with this challenge. This has led to increased use of videoconferencing (telehealth/remote consultation), internet-based support and employment of mobile phone and web-based apps as potential substitutes for face-to-face psychotherapy (17–19). Such approaches have been seen to benefit patients and healthcare workers in all areas of medicine due to the convenience of these methods, as well as the obvious associated reductions in the risk of contracting and transmitting the virus (20–23).

Although it is common for frontline medical staff and other healthcare workers to experience or witness physical and mental traumas, as well as suffering and death as part of their daily routine, such incidences increased significantly during the first waves of the pandemic. Several reviews and meta-analyses have investigated the effects of the pandemic on healthcare workers around the world, and these have identified an increased prevalence of conditions in this population, such as acute stress disorder, anxiety, burnout, depression and PTSD (24–31).

**Figure 2:**
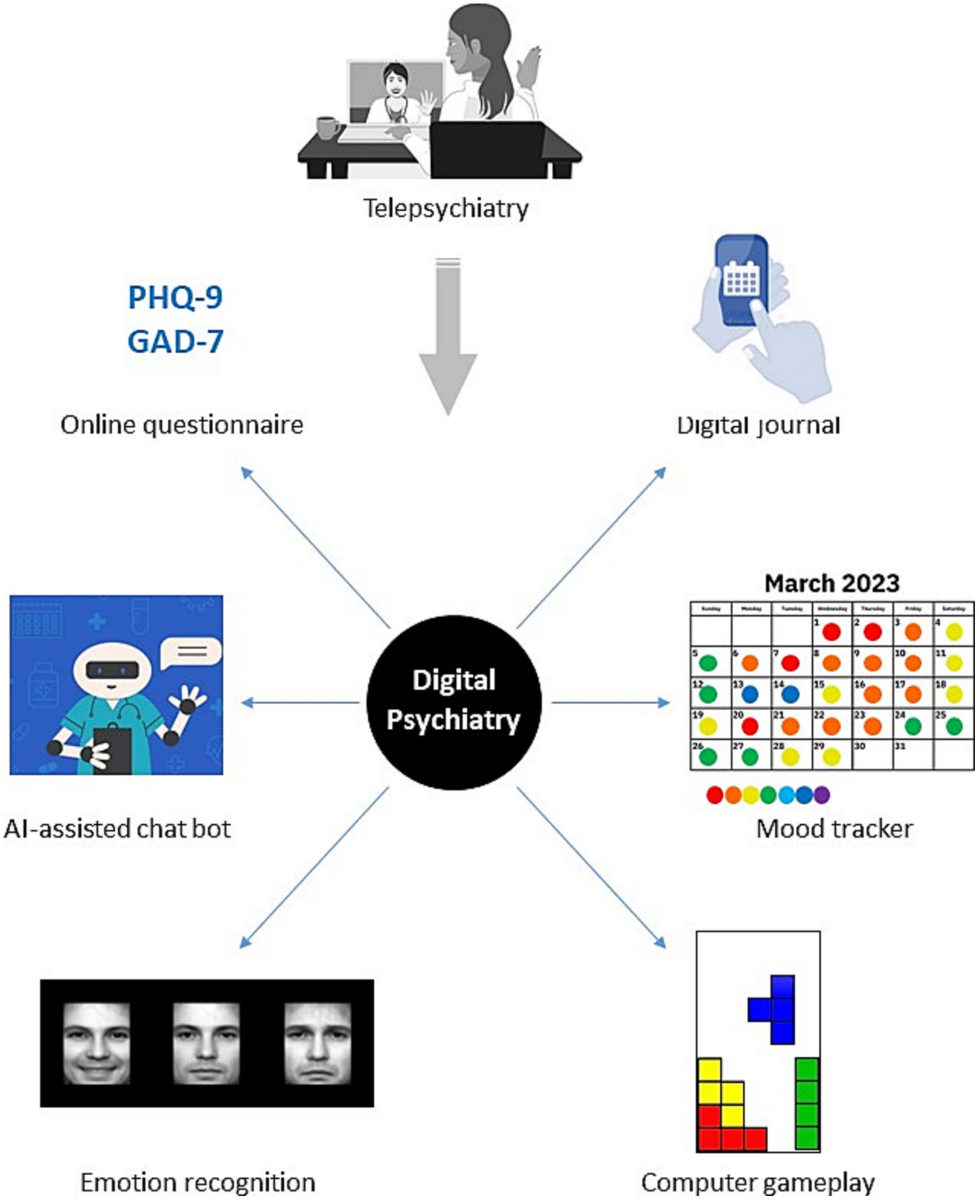
Partial overview of telecommunication and digital technologies that can be used for remote mental health care.

**Figure 3:**
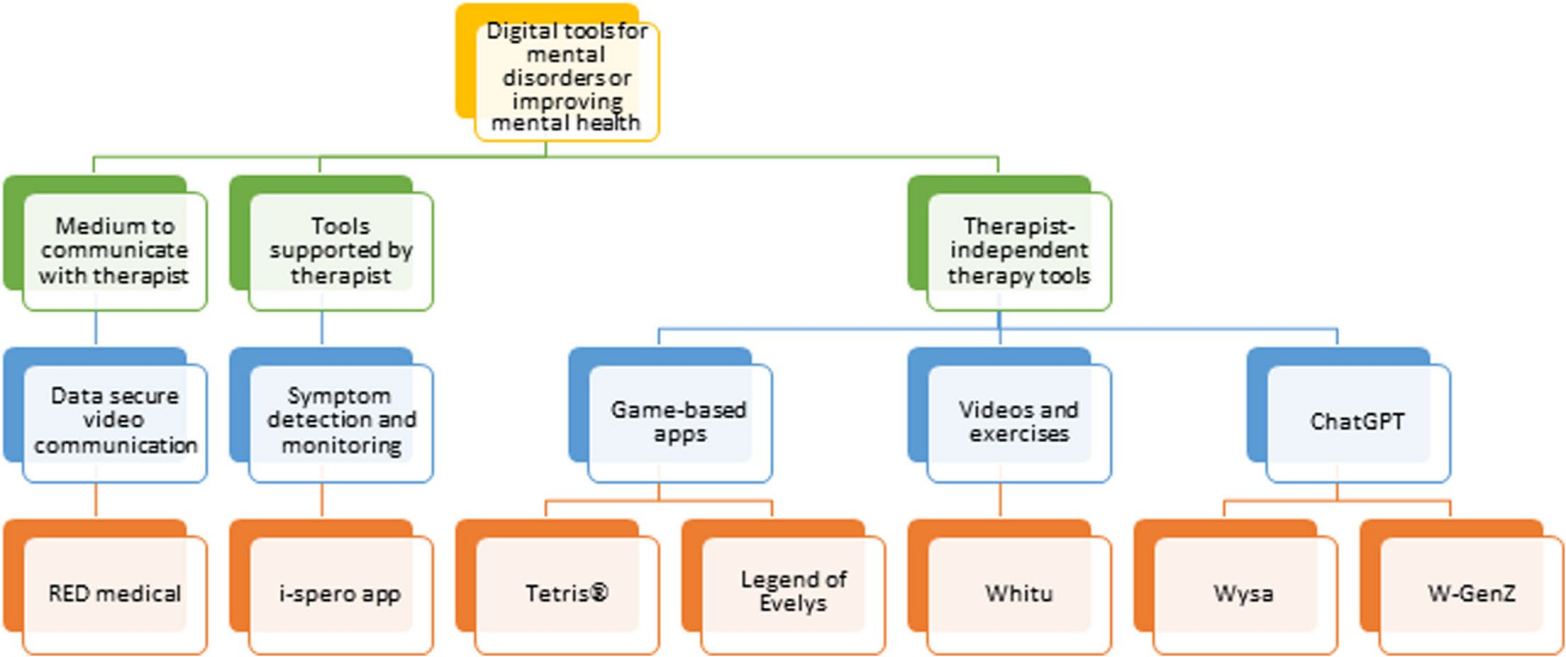
Flow chart of telecommunication technologies and digital apps in remote mental health care.

In this narrative review, we describe how digital technologies such as questionnaire-based apps, artificial intelligence (AI)-guided chatbots, online therapy software, goal-oriented physical activity apps and video games have helped to meet the challenges of the COVID-19 pandemic in delivering mental healthcare solutions (Figures 2, 3). We have focused these efforts on conditions such as depression, anxiety, stress and trauma, which markedly increased in prevalence during the pandemic (32, 33). Also, we have identified areas where the most significant progress has been made, as well as scenarios where further research is needed to achieve the most effective outcomes. Our main focus is to describe such digital approaches for use in diagnostics, patient monitoring and as a treatment option for the general public and frontline medical staff suffering with mental health issues.

## 2 Symptom monitoring and treatment guidance

### 2.1 Telepsychiatry

Telepsychiatry is defined as the remote delivery of psychiatric services through telecommunication technologies (34). It is well-established service that has been in operation for approximately seven decades launched by the Samaritans service in 1953 (35). However, telepsychiatry has now moved into a new phase through on demand provision of personalised and confidential mental health care delivery through user friendly interfaces, which have evolved to the use of digital tools to that can record, analyse and offer suggestions for behavioural or activity-based exercises as therapeutics (36). Telepsychiatry offers collaborative care models, enabling mental health professionals to work together with other healthcare providers to enhance treatment outcomes and provide comprehensive care. It also promotes continuity of care by allowing regular contact, follow-up appointments, and medication management without the need for frequent physical visits. Additionally, telepsychiatry is a cost-effective option, reducing travel expenses and making mental health care more affordable and accessible compared to in-person sessions (37). One of the video communication options used in Germany is RED medical. It not only allows data protection during the therapeutic conversation but can also be used for documentation, requesting examinations and prescribing treatment (38).

### 2.2 Digital technologies for mental health care

Many digital approaches and algorithms have been tested for purposes such as assessing symptom severity and for guiding treatment options in individuals suffering from psychiatric symptoms. To discuss these in sufficient detail is beyond the scope of this review [for more comprehensive reviews on this topic, see (39–41)]. Instead we focus on a few approaches which have been applied with mixed success during the pandemic. One means by which the use of mobile apps in mental health care may work results from the fact that they empower the patients with increased control through the ability of self-management (42). The same paper also suggested that the healthcare system would benefit as the use of these apps may establish a stronger bond with patients by providing a bridge between sessions, thereby helping patients to feel that they are not alone. In a scoping review, Ahmed et al. demonstrated the emergence of low-cost, consumer-grade biosensors as a potentially effective self-care monitoring and assessment approach for mental illnesses such as anxiety and depression (43). Most of these are wrist-worn devices such as smart bands and smart watches due to their ability to monitor habits and vital signs without the need for effort or causing any discomfort for the users.

Many of these apps were developed over several years before the pandemic had begun. For example, the PReDictive algorithm to guide antidepressant Treatment (PReDicT) was assessed between 2017 and 2019 to determine whether early changes in emotional processing during antidepressant treatment could be used to predict clinical response after one to two months of treatment (44–46). In one trial, depressed patients (*n* = 74) completed measures of emotional bias and were rated according to their other symptoms before and after 1 week of treatment and response was assessed 4–6 weeks later (45). The performance of each feature was determined by leave-one-out validation and the most robust predictors were tested in an independent patient group (*n* = 239). This revealed that key features of a facial emotion recognition test and subjective symptom measures could predict response at 77% accuracy in the training set and with 60% accuracy in an independent test set. This was significantly better than assessing baseline response rates alone. The online task was predicted to work based on the idea that brief treatment with antidepressants leads to an increase in the tendency of patients to recognise neutral facial expressions as positive (47). A second study by the same group assessed the utility of the PReDicT algorithm in a multicentre, open-label, randomised controlled trial of depressed patients (*n* = 913) to predict treatment response at the 8 weeks time point (46). This showed a non-significant improvement at week 24 in depressive symptoms (quick inventory of depressive symptoms (QIDS-SR-16) questionnaire) and a significant reduction in anxiety symptoms [generalised anxiety disorder assessment, seven-item version (GAD-7)], as well as a significantly greater improvement in functional outcome [social adjustment scale (SAS)] in the PReDicT arm, compared to the treatment as usual (TAU) group.

In a study which spanned the beginning of pandemic, Billings et al. tested the use of the web-based i-spero app (48, 49) at health centres in Faversham, Kent, UK, as a means of improving depression care. Sufferers with low mood, depression and anxiety who presented at primary care were given access to the app and asked to complete pre-assessments online using the PReDicT test, which involved completion of emotional word-based memory encoding (ECAT), recall (EREC) and face-based emotional recognition (FERT) tasks. The use of the app allowed a review of the outcomes of patient care at an individual level and alerted each patient if an action or change was needed. The monitoring aspects of the app also allowed the healthcare provider to contact the patient to discuss these options if needed. The results of the study showed that the group using the app had lower depression symptoms compared to the TAU group at the 6 months time point. However, no significant differences in symptoms of anxiety or general wellbeing were observed between the groups. Also, it should be noted that the timing of the study coincided with the initial period of COVID-19 lockdown which led to reduced walk-in visitations at the clinics. This highlighted the idea that these technologies can allow clinical assessment when access to general practice or clinical facilities is more difficult, as during a pandemic or other disaster situation.

### 2.3 Remote behavioral and physiological analysis

Given the need for remote user friendly apps during the pandemic, webcam-based online communication tools like ZOOM came into standard use for project meetings and similar activities. As a result, both the interest and the potential applications of contactless, automated analysis in areas such as lie detection (50), vital parameter monitoring (51), or pain assessment (52) have grown. Underlying techniques such as the detection of (micro)expressions (53) in combination with others modalities like gesture and body pose can be used for the early detection of depression, since depression can impair the ability to express emotions such as surprise or sadness (54).

In many areas of medicine, the so-called “diagnostic by appearance” plays a key role in diagnosis and disease monitoring, despite the availability of modern medical technology (55). This occurs especially in emergency and acute medicine. In numerous diseases and health disorders, doctors can make differential diagnostic considerations with therapeutic consequences based on externally visible pathognomonic changes. During visual diagnosis, it is important to recognize, compare, and assess factors such as the color of the skin and mucous membranes, specific/local color changes (e.g., of the sclera, bruises), facial expressions, and externally visible injuries. However, the execution of visual diagnosis is influenced by numerous objective and subjective factors. Particularly challenging and sometimes problematic factors that affect the examination and assessment of the patient include:

- Inter-individual variations in symptom manifestations.
- The absence or limited quantifiability of facial or body features.
- The necessity of patient cooperation.

This situation can be illustrated by the problem of “pain detection and quantification.” Pain causes a subjectively perceived and processed impairment in the patient and has physiological and psychological consequences. The quality and intensity of perceived pain depend on numerous influences. Both patient-dependent and patient-independent factors affect the presence and intensity of pain. To subjectively quantify pain, various methods of patient questioning, physiological parameters (heart rate, blood pressure) (51) and, to a limited extent, accompanying symptoms (e.g., nausea) can be used. Especially in patient interviews to describe the location, quality, and intensity of the perceived pain and its quantification using a Visual Analog Scale, it is necessary for the sufficiently vigilant patient to be fully cooperative. Adequate pain management is important for the further course of illness and treatment in these patients. Attempts to better quantify pain and its course in a patient-independent and practical manner have so far yielded no significant progress.

One promising approach in this regard is camera-based facial diagnostics (56). The use of camera-based facial diagnostics appears to be suitable for objectification and quantification of pain states but also for other applications. The sedative effect of administered opioid pain medications, known in pain therapy and feared as a potential side effect, leads to another potential application of camera-based facial diagnostics: vigilance monitoring. A rough qualitative description of vigilance (awake – drowsy) is possible. Progress assessments play a central role in emergency diagnostics (e.g., intracerebral bleeding), therapy monitoring (e.g., after opioid administration), and post-anesthetic observation (e.g., potential excess of anesthetic agents and/or shock states).

Similar to pain therapy, in addition to assessing measurable but nonspecific physiological parameters, the examiner relies on facial expression, gestures, facial color, sweat production, and stimulus-related reactions of the patient’s face. With the work of (56, 57), it is possible to relate the health conditions of patients and their changes to corresponding facial changes and investigate camera-based automatic analysis methods for their practicality. The focus is on pain. The reason for choosing pain as the criterion is that:

- Pain management measures are routinely applied in the recovery room.
- Patients are under constant equipment and personnel supervision.
- Pain exhibits a particularly pronounced facial reflection.

Thus, the evidence (52, 56, 57) shows that the proposed camera-based facial diagnostic system can diagnose these health conditions. A wide application for recognition of health status from the human face using camera-based pattern recognition apps combined with AI is made possible as a result.

### 2.4 Chat- and psychoeducation-based apps

A 12 weeks randomized controlled trial during the pandemic evaluated the feasibility and efficacy of using a natural language and machine learning chat-based app (W-GenZ) for mood tracking and for facilitating tailored intervention in 13 to 17 years-olds with moderate symptoms of depression (58). The outcome of the study revealed that use of the app was feasible and acceptable to both the teenagers and their parents, and depressive symptoms in the young participants showed a shift from the moderate to mild category by 4 weeks in the treatment arm compared to the control group, as determined using Patient Health Questionnaire (PHQ-9) scores. Similarly, a prospective randomised controlled trial carried out in New Zealand evaluated the efficacy and acceptability of a well-being app during the pandemic (59). The app (Whitu) contained elements aimed at helping young individuals to evaluate their emotions, adopt relaxation and mindfulness practices, learn self-compassion and gratitude, form inter-person connections, and to focus on physical health and goalsetting. The results revealed that individuals in the Whitu arm showed significantly higher emotional and mental well-being, selfcompassion and sleep with significantly lower depression (Centre for Epidemiological Studies Depression Scale), anxiety (GAD-7 scale) and stress (10-item Perceived Stress Scale) symptoms, compared with the controls at both 4 weeks and 3 months.

A randomized control trial which tested the efficacy of a mobile phone-based app (Foundations) in adults during the first wave of the pandemic in the UK found significant improvements in anxiety (GAD-7), resilience (10-item Connor-Davidson Resilience scale), sleep (Minimal Insomnia Scale) and mental well-being (WHO-5) scores within 2 weeks compared to the control group (60). Further improvements in all of these scores were noted after 4 weeks. Sinha et al. carried out a retrospective observational study on use and efficacy of an artificial intelligence (AI)-guided mental health app called Wysa from March 2020 to October 2021 in three countries [United States of America (USA), UK, India] (61). They found a significant positive correlation between the increase in installations of the Wysa app and COVID-19 cases in the UK and India, with higher engagement compared to the pre-pandemic period. In addition, depression and anxiety symptoms showed significant improvements as determined using PHQ-9 and GAD-7 assessments, respectively.

Another online therapy-based app which may be useful in disaster situations like the pandemic is a called Deprexis^®^, which is both Conformite Europeenne (CE) marked and Food and Drug Administration (FDA) approved and can be prescribed to patients by their psychiatrist (62, 63). Deprexis provides an online support system through its capacity to recognise negative thinking patterns. It can also help the patient to learn new behaviours and integrate these into their everyday life based on the principals of cognitive behavioural therapy. The programme uses a specific dialogue with each patient and offers instructions on therapeutic exercises through e-mail and/or short message service (SMS) contacts. The aim is to teach the patient techniques that he/she can apply to him/herself in order to overcome depression on his/her own.

#### 2.4.1 Chat GPT

ChatGPT, introduced in November 2022, is expected to have a significant impact on a range of industries, including healthcare, medical education, biomedical research, and scientific writing (64). Large Language Models such as ChatGPT could enhance user engagement in health care apps by providing a conversational interface and personalized responses, leading to more natural and effective communication (65). According to a publication by Patel and Lam et al., ChatGPT delivers tailored responses in a conversational style, even recalling prior interactions. They stated that ChatGPT exhibits impressive performance in various applications. It was able to write satisfactory discharge summaries, having the potential to increase productivity by physicians (66).

The effectiveness of ChatGPT in providing support in cases of anxiety and depression has been assessed based on cross-questions and responses (67). This revealed inconsistencies and low reliability of the chatbot for this purpose. Being a generative model ChatGPT’s output may contain inaccuracies or biases, called hallucinations (65). This might include citing non-existent article references or perpetuating sexist stereotypes. The program could also respond to harmful instructions, like giving instructions on how to steal apples. Although OpenAI established safety measures to reduce these risks, users have found ways to bypass them. Moreover, as ChatGPT’s output might be used to train future versions of the model, the errors could be repeated and amplified (68).

However, it is crucial to report on safety and bias protection mechanisms to mitigate potential harm to users, as well as regularly monitor and track these mechanisms. There are also some concerns about the use of large language models like ChatGPT, as the accuracy and integrity of the texts generated by these models is unknown (69).

### 2.5 Digital healthcare devices recommended for use in the United Kingdom, United States of America, and Germany

This section highlights digital technologies which have been approved by medical agencies in various countries but still require assessment in bridging the mental healthcare gap in times of inaccessibility such as during pandemic situations.

In the UK (October, 2022), the National Institute for Health and Care Excellence (NICE) and the Medicines and Healthcare Products Regulatory Agency (MHRA) were awarded £1.8 m GBP by the Wellcome Trust to develop a regulatory framework for use of digital mental health tools as medical devices (70). On March 1 2023, NICE issued a conditional recommendation for digital enabled therapies to treat depression and anxiety disorders in adults (Table 1) (71). The use of each device involves cognitive behavioural therapy via a National Health Service (NHS) Talking Therapies clinician and each must be approved by the appropriate regulatory authority [Digital Technology Assessment Criteria (NHS England), CE or UK Conformity Assessed (UKCA) marking] and comply with NHS Talking Therapies digitally enabled therapies.

**Table 1:** Devices conditionally recommended by NICE in the UK for treatment of depression and anxiety disorders.

Device	Diagnosis	Complementary therapies
Perspectives	Body dysmorphic disorder	High intensity therapist
Beating the blues	Generalised anxiety symptoms	Psychological wellbeing practitioner
Space from anxiety (SilverCloud)	Generalised anxiety symptoms	Psychological wellbeing practitioner
iCT-PTSD	Post-traumatic stress disorder	High intensity therapist
Spring	Post-traumatic stress disorder	High intensity therapist
iCT-SAD	Social anxiety disorder	High intensity therapist
Deprexis	Depression	Psychological wellbeing practitioner
Beating the blues	Depression	Psychological wellbeing practitioner
Space from anxiety (SilverCloud)	Depression	Psychological wellbeing practitioner

In the USA, four devices have been marketed for treatment of depression or anxiety (72). These are: (1) Flow Neuroscience which uses transcranial Direct Current Stimulation (tDCS) to stimulate neuronal firing in the dorsolateral prefrontal cortex; (2) Neurolief Relivion which uses the same approach as Flow Neuroscience but targets occipital and trigeminal nerves; (3) Cervella which uses Cranial Electrotherapy Stimulation (CES) via conductive electrodes in the ear pads of noise-cancelling headphones to promote sleep; and (4) Alpha-Stim which uses the same approach as Cervella but with clips that fasten to the earlobes. However, the only device on the list with FDA approval for treatment of depression, anxiety and sleep disorder is Cervella.

A search of the Federal Institute for Drugs and Medical Devices (Bundesinstitut für Arzneimittel und Medizinprodukte, BfArM) showed that there are 18 apps which have been approved for use by health insurance companies with applications in various psychiatric conditions in Germany (Table 2) (73).

**Table 2:** Devices conditionally recommended by digital health applications in Germany for treatment of mental disorders.

Device	Diagnosis	Supported by
Deprexis	Depression	General practitioner, psychiatrist or psychotherapist
Edupression	Depression	Psychological wellbeing practitioner
Elona therapy depression	Depression	Psychiatrist or psychotherapist
HelloBetter Panik	Anxiety disorder	Psychological wellbeing practitioner
HelloBetter Schlafen	Sleeping disorder	Psychological wellbeing practitioner
HelloBetter Stress and Burnout	Stress, burnout	Psychotherapist
Invirto	Anxiety disorder	Psychiatrist or psychotherapist
Mindable	Anxiety disorder	Psychological wellbeing practitioner
My7stepsApp	Depression	Psychological wellbeing practitioner
NichtraucherHelden-App	Nicotine dependence	Without supportive therapy
Novego: Ängste überwinden	Anxiety disorder	Psychiatrist or psychotherapist
Novego: Depressionen bewältigen	Depression	Psychiatrist or psychotherapist
Priovi	Borderline personality disorder	Psychiatrist or psychotherapist
Selfapys Online-Kurs bei Binge-Eating-Störung	Eating disorder	Psychological wellbeing practitioner
Selfapys Online-Kurs bei Bulimia Nervosa	Eating disorder	Psychological wellbeing practitioner
Selfapys Online-Kurs bei Depression	Depression	Psychological wellbeing practitioner
Selfapys Online-Kurs bei Generalisierter Angststörung	Generalised anxiety symptoms	Psychological wellbeing practitioner
Smoke Free	Nicotine dependence	Without supportive therapy
Somnio	Sleeping disorder	Physician or psychotherapist
Velibra	Anxiety disorder	General practitioner, psychiatrist or psychotherapist
Vorvida	Substance abuse disorder	Physician
Zanadio	Eating disorder: obesity	Physician or psychotherapist

## 3 Physical activity and game-based apps

Online physical activity sessions, such as virtual reality exercise (74), interactive physical-activity-based games (75), singing (76), and dance (77), have also been shown to be effective in reducing depressive and anxious symptoms.

Groenveld et al. investigated the utility of self-administered virtual reality physical, relaxation and cognitive exercises to aid physiotherapy-based recovery after individuals had been infected with the SARS-CoV-2 virus (74). All participants performed the exercises for 6 weeks and physical performance, activities, cognitive function, quality of life, and anxiety and depression symptoms were measured before and after this interval. The results showed that 75% of the patients found that the experience had a positive effect on their recovery from the viral infection, with an average use time of 30 min per session, and frequency of 3 to 4 times per week, over 3–6 weeks. Although adherence to the physical-based exercises decreased over time, the use of the relaxation and cognition-based exercises was constant throughout the study period. Importantly, the physical performance and quality of life measures showed significant improvements after the 6 weeks session, compared to the levels seen at baseline time point. Similarly, Kim et al. described a framework for use of gamification in the development of exercise-based games focussed on cardiovascular and strength training as a treatment for depression (75). The researchers are currently planning a pilot study to obtain user feedback to guide refinement of the game content. This will be followed by a randomized controlled trial to assess the efficacy and cost-effectiveness of this intervention compared existing standard methods.

Exploring other avenues, a 12 weeks randomised controlled trial assessed the effect of singing compared to usual care on quality of life, breathlessness, balance confidence, physical activity, as well as depression (PHQ-9) and anxiety (GAD-7) in 120 chronic obstructive pulmonary disease (COPD) patients (76). As nine singing and nine control patients had completed five face-to-face sessions and seven online sessions due to adjustments made for COVID-19 restrictions, the authors reported on the outcomes in this group as a pilot study to inform further trials. The results revealed improvements in depression and balance confidence scores in this group that transitioned to the online sessions, despite a general preference for face-to-face meetings. In another study, Humphries et al. found that a single 60 min session of self-selected dance resulted in an increase in positive affect and self-esteem and a decrease in negative affect and depression symptoms in healthy adults (*n* = 47) during the social isolation associated with the COVID-19 pandemic (77).

Tong et al. investigated several aspects regarding the use of mobile apps and fitness trackers during the pandemic using an online survey of 552 adults from June to September 2020 in Australia (78). This showed that users of fitness trackers and health-related apps were more physically active compared to non-users and more women used these health-related apps compared to men. Interestingly, most users stated that the apps did not adapt quickly enough to keep pace with the changing conditions of the pandemic. This highlights a potential problem that should be addressed quickly in future disaster situations.

A recent systematic review found that playing video games helped many people from all aspects of life to cope with the difficult life experiences that they encountered during the pandemic, particularly the times of the peak lockdown and isolation periods (79). Interestingly, this revealed that augmented reality and online multiplayer games helped to ameliorate the loneliness, stress and anxiety experienced by adolescents and young adults during these times. It is possible that the feeling of regaining control over life and social contacts, even if this is only virtual, plays a role in this. On the other hand, playing such games had detrimental addictive effects in the longer term for high-risk individuals, such as problematic gamers. A collaborative study carried out by the Centre for Addiction and Mental Health and National Research Council of Canada evaluated an online cognitive behavioural therapy-based video game called Legend of Evelys for ameliorating the increased stress due to the pandemic (80). The storyline of this fantasy game was based on the emotional status of people in a fictional setting that mirrored the negative effects of the pandemic on the population. The game allowed interactions between the avatars chosen by the users with prompts encouraging their engagement in therapeutic behavioural exercises. After a 2 weeks trial the participants gave the game a rating of “good” (Systems Usability Scale score) and the study showed a significant reduction in perceived stress compared to the baseline levels.

## 4 Treatment of frontline medical staff

A number of recent studies have demonstrated that frontline medical professionals and healthcare staff experienced increased stress, PTSD, anxiety, and depression at peak periods of the COVID-19 pandemic. There are many reasons why these stressors may have occurred. On the one hand, there was not enough previous experience, which meant that there were no guidelines to give support and security. On the other hand, due to the high number of patients, triage was necessary and not all patients could be offered help due to limited number of ventilators and nurses. This can stress medical staff to a great extent when there is the impression that help could potentially be given but resources are not sufficient, and the feeling of helpless overload sets in. This contradicts the professional ethos of many clinical staff members.

Intrusive memories are key feature of post-traumatic stress syndrome (PTSD) (81). Interventions within just a few hours after the initial trauma have shown some effectiveness in blocking the encoding or consolidation of these memories into long-term storage (82, 83). It is also now considered that even consolidated memories can become labile, albeit transiently, if they are recalled into working memory (84). This suggests that interventions for long-term intrusive memories may also be effective. One key question is: what sort of intervention would be most effective and could be translated easily into routine practice with minimal discomfort to the sufferers?

In 2017, Hagenaars et al. tested whether memory reactivation 4 days after participants watched an aversive film would result in fewer intrusive memories recorded in a diary if the participants performed a visuospatial or verbal task, compared to a no-task control (85). The film consisted of live footage of road traffic accidents and ensuing traumas such as the work of the emergency recovery personnel and the effects on the victims, including injuries and death. The visuospatial task consisted of playing the computer game Tetris^®^ (86) and the verbal task consisted of word games, each performed for 10 min. The results showed that both the visuospatial and verbal tasks were effective in the reducing the number of intrusive memories. Interestingly, the verbal task was marginally more effective in reducing intrusive memories but was rated by the participants as more difficult to perform, compared to the visuospatial task.

In 2018, the same research group extended these studies by carrying out a randomised controlled trial to investigate the effects of the visuospatial intervention for 20 min per day for 1 week on the number of intrusive memories experienced by real-life trauma victims (87). They found that there were fewer intrusive memories in the Tetris-play group compared to a control group that recorded an activity log for the same 20 min time period. The participants in the Tetris group also reported less distress from the intrusive memories at 1 week, as determined using the Impact of Event Scale-Revised (88). Members of the same research group also carried out a single case series study involving 20 longstanding PTSD patients, who were administered a similar Tetris gameplay intervention for 25 min on a weekly basis over 5 to 10 weeks in an inpatient setting (89). The results showed that intrusive memories decreased by an average 64% and 16 out of the 20 participants met the criteria for showing a favourable response to the intervention. Similar positive findings were reported using this same intervention in the case of refugees with traumatic memory intrusions in Stockholm Sweden (90) as well as for trauma-exposed women in Iceland (91).

Two studies were registered on clinicaltrials.gov to investigate the effects of applying the remotely delivered Tetris gameplay intervention to reduce the number of intrusive memories in frontline healthcare staff who experienced or witnessed traumas involving their work during the COVID-19 pandemic. The first of these aims to investigate the effects of a remotely delivered cognitive task over 4 weeks on intrusive memories and other symptoms in Swedish hospital staff (92, 93). The intervention consists of a brief memory cue of the traumatic event followed by Tetris gameplay. The control arm consisted of staff who listened to a daily podcast for the same time-period. The design includes completion of a daily intrusive memory diary and various questionnaires on day 1, week 1, week 4 and various time points up to 6 months, as possible. It is expected that the results of the study will be reported in 2023. The second study used a similar design to reduce the number of intrusive memories experienced by National Health Service intensive care unit staff who worked during the COVID-19 pandemic in the United Kingdom (94). A key difference between this and the Swedish study was the incorporation of a delayed arm in which the participants were given usual care for the first 4 weeks followed by access to the intervention for the next 4 weeks. The primary outcomes in this study were the number of intrusive memories at week 4 in both arms of the study. The secondary outcomes included the number of intrusive memories at week 8 compared to week 4 in the delayed arm. The results of this study were published recently in Molecular Psychiatry (95). The results confirmed a positive treatment effect, with the immediate arm reporting fewer intrusive memories compared to the delayed arm at week 4. The results of the delayed arm of the study will be published later this year.

## 5 Limitations and current challenges

As with all narrative reviews, this paper was limited by incompleteness of the literature search. Such completeness would not be possible in this case as we have focussed on a wide variety of digital healthcare technologies and apps which have either emerged or showed increased usage during the pandemic. Furthermore, the efficacy of these approaches have been investigated in a number of psychiatric disease areas, and so we opted to limit this to investigation of depression, anxiety, stress and trauma, which all increased in occurrence during the first waves of the COVID-19 infections. In addition, healthcare apps offer many benefits to users but they also have limitations and current challenges that need to be addressed. One significant challenge is user engagement, where some people may download a healthcare app but fail to consistently use it due to lack of motivation, forgetfulness, or poor user experience. One study found that although the number of app installs and daily active minutes of use may seem high, only a small portion of users actually used the apps for a long period of time (96). In particular, elderly people were reported to feel discomfort integrating health apps and wearables into their routine due to the constant reminder of their illness, according to qualitative interviews (97). Therefore, the usability and acceptability of digital health apps need to be improved for people with chronic conditions (98).

Additionally, there is a risk of over-reliance on healthcare apps, which might be racially biased due to the choice of distinct population groups used to train the relevant algorithms (99), or they may be unable to properly address crises like suicidal ideation due to inconsistencies in language across different apps (100). Also, there is the problem of the digital divide due to issues regarding access to the internet or technologies for some individuals. This is particularly difficult for those who are already socially or economically disadvantaged, the elderly population and those with cognitive disorders, which can further perpetuate inequities (101–103). To add to the problem, private and governmental organizations and stakeholders may encounter challenges in adequately validating and approving new digital health technologies. Therefore, more research is necessary before implementing a digital product in the healthcare sector (104). There are also concerns about data security and privacy risks, as highlighted by various studies, and governmental regulations are needed to enhance fairness and protection of personal data (105).

Health care apps need to be systematically assessed to ensure the safety of target users (106). With the evolution of healthcare apps, there are new risks associated with the automation of the chat interface. App creators need to report on safety and bias protection mechanisms to mitigate potential harm to their users, explain potential risks and harms to users, and regularly monitor and track these mechanisms (106).

## 6 What next steps are needed?

The COVID-19 pandemic has provided valuable insights into the role of digital mental healthcare and highlighted key areas that should be integrated into preparedness plans to help us cope better in case of future pandemics and other crises. The lessons learned include the importance of flexibility, data security, and the need for interdisciplinary collaboration, along with enabling access to technology for diverse populations, including those with limited access or for individuals who may face barriers due to socioeconomic and other factors (Table 3). This information can be used to inform future pandemic preparedness plans and to help ensure that mental health support is readily available during challenging times. This will require addressing key areas including the need for scalability, accessibility, adaptability, as well as instilling an increased awareness of mental health issues during crisis situations and the importance of early intervention to maximize treatment effectiveness (Table 4). This will help to make sure that digital mental health tools are safe, effective, and accessible to those who need them.

**Table 3:** Insights into digital mental health needs highlighted by the COVID-19 pandemic.

Area identified	Description
Telehealth and teletherapy	The pandemic underscored the importance of telehealth and teletherapy as crucial tools for delivering mental health care during times when in-person visits are restricted
Scalability and accessibility	Individuals should have equitable access to technologies such as smartphones, computers, and internet connectivity to address the digital divide
Flexibility and adaptability	Flexibility and adaptability are needed to respond rapidly to changing circumstances, including necessity of shifting to remote care and adjusting treatment plans as appropriate
Crisis response/helpline support	Crisis helplines and digital mental health crisis response tools should be well-prepared to handle increased demand and provide timely support to those in need
Mental health awareness	Raising awareness on importance of mental health and reducing stigma should be a continuous effort to help those in need recognize signs of distress and access available support
Need for early intervention	Digital mental health tools can be used to help identify individuals at risk and provide support before symptoms worsen
Policy/funding support	Governments and healthcare systems should allocate resources, create policies, and provide funding to support development, implementation, and upkeep of digital services
International sharing	International collaboration and sharing of best practices in digital mental healthcare can help nations learn from each other and improve preparedness for future pandemics

**Table 4:** Areas that should be addressed next to promote successful integration of digital mental health interventions into clinical practice.

Area of need	Description
Evidence-based interventions	Continued research and large-scale, well-designed studies are needed to establish and validate effectiveness of digital mental health interventions
Interoperability	Seamless information sharing and coordination of care should be promoted between digital mental health tools and healthcare systems, including electronic records
Telehealth integration	Digital mental health services should be integrated with broader telehealth infrastructure to make it easier for patients to access remote care when needed
International collaboration	Collaboration and knowledge sharing should be fostered among researchers, clinicians and technology developers on a global scale to accelerate progress in digital psychiatry
Training and education	Mental health professionals should be trained in the use of digital tools and teletherapy techniques
Scalability and accessibility	Digital mental health solutions should be made scalable and accessible to diverse populations, including those with limited or hindered access to technology
User-centered design	Patients and end-users should be involved in the design and development process to create user-friendly and engaging digital mental health solutions
Personalization and tailoring	Algorithms and AI systems should be developed and refined to enable personalize treatment plans based on individual patient needs, abilities and preferences
Integration of peer support	Peer support networks and community-based resources should be integrated into digital technologies to enhance engagement and social support
Data privacy and security	Regulatory compliance and secure data storage should be strengthened in digital mental health platforms to protect patient information
Ethical considerations	Ethical guidelines should be established and updated as needed to address issues such as informed consent, privacy, and responsible use of AI in mental health care
Long-term monitoring	Develop strategies for long-term monitoring and follow-up using digital tools to support ongoing mental health care and relapse prevention

## 7 Conclusions and future perspectives

The evidence presented in this review supports the case that use of digital technologies such as mobile phone apps, chat bots and video games, may serve as an effective coping strategy with therapeutic potential during epidemic, pandemic or disaster situations. Several studies have now demonstrated that such approaches not only enabled patients with mental disorders to effectively maintain their visitations with their healthcare providers, in many cases they were also shown to be effective in ameliorating symptoms such as stress, anxiety and depression. For example, the use of online mood-tracking apps, questionnaires and AI-guided chat bots appears to support patients by involving them in monitoring their own symptoms, which can be used to inform the treatment process.

As further examples, several studies have demonstrated the effectiveness of online guided physical activities, such as physical fitness exercises, dancing and singing. Computer or mobile phone-based games have also proven to be a useful distraction. Along these lines, playing the computer game Tetris has been shown to alleviate the number of intrusive memories in individuals who have either witnessed or experienced traumas such as terrible or fatal injuries of road traffic accident victims. Considering the disastrous effects of the COVID-19 pandemic on mental health issues on a global level, it will important to incorporate many of digital approaches into the rapid response infrastructure to help humanity to cope more effectively at both the physical and mental levels with disaster scenarios. This includes earthquakes, floods, tornados, hurricanes and future pandemics. Nevertheless, it is crucial to continue researching ways to enhance the user experience in order to ensure the successful integration of digital mental health interventions into clinical practice and promote increased user engagement in order to further increase therapeutic effectiveness of these programs.

## Author contributions

PG, JS, VV, and JE wrote and edited the manuscript. PF proofread and provided valuable input into the final version. All authors contributed to the article and approved the submitted version.
